# *Phaseolus vulgaris* L. Extract: Alpha-Amylase Inhibition against Metabolic Syndrome in Mice

**DOI:** 10.3390/nu11081778

**Published:** 2019-08-01

**Authors:** Laura Micheli, Elena Lucarini, Elena Trallori, Carmen Avagliano, Carmen De Caro, Roberto Russo, Antonio Calignano, Carla Ghelardini, Alessandra Pacini, Lorenzo Di Cesare Mannelli

**Affiliations:** 1 Department of Neuroscience, Psychology, Drug Research and Child Health-Neurofarba-Pharmacology and Toxicology Section, University of Florence, 50139 Florence, Italy; 2 Department of Pharmacy, University of Naples “Federico II” Naples, 80131 Naples, Italy; 3 Department of Science of Health, School of Medicine and Surgery, University of Catanzaro, 88100 Catanzaro, Italy; 4 Department of Experimental and Clinical Medicine, Anatomy and Histology Section, University of Florence, 50134 Florence, Italy

**Keywords:** *P. vulgaris* extract, metabolic syndrome, HFD, alpha-amylase inhibitor

## Abstract

To examine the effects of the alpha-amylase inhibitor isoform 1 called phaseolamin, a standardized extract from white kidney beans (*Phaseolus vulgaris* L.) was tested against the hallmarks of metabolic syndrome. The efficacy of a *per os* repeated treatment with *P. vulgaris* extract (500 mg/kg) was compared with metformin (100 mg/kg) and atorvastatin (10 mg/kg) in a model of metabolic syndrome evoked by prolonged high fat diet (HFD; week 1 to week 19) in C57BL/6 mice. Bean extract and compounds administration started after metabolic syndrome establishment (week 11). *P. vulgaris* extract reduced the body weight overtime, as well as effectively lowered glycaemia, triglycerides, and cholesterol. On week 19, bean extract normalized the HFD-evoked tolerance to glucose and insulin. According to the phytochemical characterization, it inhibited the alpha-amylase activity. Animals treated with the extract were rescued from motor impairments and nociceptive threshold alterations induced by HFD. Specific organs analysis revealed that *P. vulgaris* extract decreased hepatic steatosis and lipid peroxidation in liver. It protected the heart from HFD oxidative alterations increasing the expression of the detoxifying enzymes catalase and glutathione reductase, and normalizing NADH dehydrogenase level. The histological analysis of aorta showed a protection about the development of fatty streaks in the muscular layers. In conclusion, a prolonged treatment with the standardized extract of *P. vulgaris* significantly reduced several pathological features related to a metabolic syndrome-like condition; a multifactorial approach that candidates this vegetal product as a possible therapeutic option against metabolic syndrome.

## 1. Introduction

Metabolic syndrome was first described by [1], who named it “Syndrome X”. Since then, the interest from the investigators has increased constantly, and many were the names given to it, e.g., the insulin-resistance syndrome, the hypertriglyceridemic waist, and metabolic syndrome. The latter, the most used term in the cardiovascular field, will be used in this article to refer to this syndrome [2]. 

The syndrome is a group of co-occurring and interconnected biochemical and metabolic disorders, among which the hallmarks are altered glucose metabolism, insulin resistance, dyslipidemia, hypertension, abdominal obesity, hepatic steatosis, and hypercholesterolemia. The modern lifestyle, defined by over-nutrition and very little physical exercise, has recently increased its incidence. The array of disorders which compose metabolic syndrome often represent the preparatory soil for type 2 diabetes and atherogenic cardiovascular diseases [2,3,4]. 

Treatment strategies for metabolic syndrome could involve the use of nutraceuticals, most of which have plant origins (phytochemicals) associated with lifestyle improvement [5,6].

The pancreatic alpha-amylase inhibitor isoform 1 (alpha-AI 1), also known as phaseolamin, is a constituent protein of the common white and kidney beans (*Phaseolus vulgaris* L.). Next to it, there are two other isoforms and an alpha-amylase inhibitor like (alpha-AI 2, alpha-AI 3 and alpha-AIL, respectively). The most widely distributed in the plant is the alpha-AI 1, which represents about one tenth of the total seed protein content [7]. This enzyme hampers the activity of some mammalian and insect alpha-amylase, but it does not affect the plant endogenous enzyme. Interestingly, each variety of *P. vulgaris* produces an alpha-AI type 1 with a fairly different primary structure; nevertheless, all the enzymes show the same inhibitory activity on animal amylases [8]. This enzyme was discovered in 1945 by [9], and was characterized and named “phaseolamin” in 1975 by [10]. Since then, phaseolamin was tested for its efficacy as a starch blocker and purified extracts were used as human dietary supplements with anti-hyperglycaemic and anti-obesity purpose [11]. The results were not as expected, probably because of an insufficient inhibitory activity on human alpha-amylase. The successive progresses made in the protein extraction, purification, and standardization fields have allowed the production of bean plant extracts with higher and more effective inhibitory activity on carbohydrate metabolism [7]. 

Moreover, although *P. vulgaris* is not the only plant to contain alpha-amylase inhibitors, as some cereal plants also produce them, there is no report about severe side effects correlated to its alpha-AIs, while the cereal-derived isoforms are associated with dermatitis and asthma [7]. 

The aim of the present study was to evaluate the efficacy of a chronic treatment with a standardized seed extract from *P. vulgaris*, containing 6% of the non-nutritive bioactive compound phaseolamin on a murine model of metabolic syndrome to compare its efficacy to a chronic treatment with two reference drugs for hyperglycaemia and hypercholesterolemia—i.e., metformin and atorvastatin, respectively. The metabolic disorders were induced by feeding with a high fat diet (HFD), containing 60% fats out of total calories, and the results were compared with those obtained from mice fed with normal diet. HFD-fed animals, indeed, reached the pathological state after 11 weeks of enriched alimentation, as confirmed by remarkably altered blood levels of glucose, triglycerides, and cholesterol. Further ex vivo analysis completed the investigation on the grade of regression of the disease reached, following each treatment. 

## 2. Materials and Methods 

### 2.1. Animals 

Male C57BL/6 mice (Envigo, Varese, Italy) weighing approximately 20 g at the beginning of the experimental procedure were used. Animals were used in Ce.S.A.L (Centro Stabulazione Animali da Laboratorio, University of Florence) and used at least one week after their arrival. Twelve mice were housed per cage (size 26 × 41 cm), kept at 23.0 ± 1.0 °C with a 12 h light/dark cycle, with lights on at 7 a.m.; during acclimatization they were fed a standard laboratory diet and tap water ad libitum. 

All animal manipulations were carried out according to the Directive 2010/63/EU of the European parliament and of the European Union council (22 September 2010) on the protection of animals used for scientific purposes. The ethical policy of the University of Florence complies with the Guide for the Care and Use of Laboratory Animals of the US National Institutes of Health (NIH Publication no. 85–23, revised 1996; University of Florence assurance number: A5278-01). Formal approval to conduct the experiments described was obtained from the Animal Subjects Review Board of the University of Florence. Experiments involving animals have been reported according to ARRIVE guidelines [12]. All efforts were made to minimize animal suffering and to reduce the number of animals used. 

### 2.2. Induction of Metabolic Syndrome

Metabolic syndrome was induced by feeding the animals with a high fat diet (HFD; Research Diets, New Brunswick, NJ) for 19 weeks ad libitum. The model is consistent, with minor modification, to what was previously published [13,14,15]. The HFD contained 60% fat, 20% protein, and 20% carbohydrate, as a percentage of total Kcal [16]. Control animals were fed ad libitum for 19 weeks with a standard diet (24% protein, 58% carbohydrate, 18% fat; Envigo, Varese, Italy).

### 2.3. Extract Preparation

The vegetal extract used in the present study (Beanblock^®^; Indena S.p.A., Milan, Italy) is a standardized dry extract containing an alpha-amylase inhibitor and phytohaemagglutinin. Briefly, *P. vulgaris* dry extract was prepared by means of aqueous extraction and alcoholic precipitation from the common kidney bean (*P. vulgaris*). Bean extract was obtained by extraction with citrate buffer and precipitation with ethanol. The extract is characterized by a standardized composition of 8.5% (*w*/*w*) tested α-amylase inhibitor, with inhibiting activity = 1400 U/mg, and phytohaemagglutinin (haemagglutinating activity 16 haemagglutinating units/mg). The manufacturing process is described in detail by [17,18]. 

### 2.4. Treatments

*P. vulgaris* dry extract 500 mg kg^−1^ (Indena S.p.A.), metformin 100 mg kg^−1^ (Carbosynth, UK), and atorvastatin 10 mg kg^−1^ (Carbosynth, UK) were suspended in 1% carboxymethylcellulose sodium salt (CMC; Sigma-Aldrich) and daily *per os* administered 30 min before the dark phase of the circadian light/dark cycle in the animal facility from week 11 until week 19. The doses of *P. vulgaris*, metformin, and atorvastatin were chosen on the basis of the literature [18,19,20]. 

### 2.5. Body Weight and Food Intake 

During the experiment, the body weight of mice was measured every 3 days, while the food intake was measured weekly. 

### 2.6. Collection of Blood and Analytical Methods

Blood was collected from the facial vein of fed mice under light ether anaesthesia into eppendorf tubes containing heparin (20 μL, 25,000 IU/5 mL) twice a month, starting from week. Samples were analyzed for glucose, triglycerides, and total cholesterol levels using a Reflotron reflectance photometric analyser (Reflotron, Roche Diagnostics and Vitros, Johnson & Johnson). Low-density lipoprotein (LDL), high-density lipoprotein (HDL) cholesterol, and alpha-amylase activity were measured using the same method only in week 19. Plasmatic levels of ghrelin and insulin were measured in week 19 using ELISA kit (Wide range mouse insulin immunoassay kit, Biorbyt; Mouse ghrelin enzyme immunoassay kit, Biorbyt). Alpha-amylase activity was measured. 

### 2.7. Glucose and Insulin Tolerance Tests

On week 19, the glucose tolerance test (GTT) and the insulin tolerance test (ITT) were performed by intraperitoneal injections of glucose (1 mg kg^−1^) and insulin (1 U kg^−1^; Humalog, Lilly) on fed animals, respectively. Blood glucose was measured by Accu-Check Aviva at 0 min, 15 min, and 30 min after glucose injections for GTT, and at 0 min, 30 min, and 60 min after insulin injection for ITT. 

### 2.8. Behavioral Tests

On week 19, the motor coordination was measured by the Rota rod test according to [21]. Mechanical allodynia was measured by Von Frey test as described by [22]. TheHargreaves’ Plantar test was used to measure the thermal nociceptive threshold as described by [23]. To evaluate anxiety-related behavior, the Elevated plus maze test was conducted as previously described by [24,25]. More details in the Supplementary Materials.

### 2.9. Tissues Explant

On week 19, after the behavioral measurements and the biochemical analysis, mice were sacrificed by cervical dislocation and tissues explant was performed. In particular, brain, liver, heart, aorta, epidydimal white adipose tissue, and kidney were collected.

### 2.10. Liver and Kidney Histopathology

Samples were fixed in 4% formalin and processed for embedding and sectioning. More details in the Supplementary Materials. 

### 2.11. Analysis of Lipid Deposition in the Arterial Wall

The aortas were washed with phosphate-buffered saline (PBS) and then fixed with 4% paraformaldehyde overnight. The proximal aorta attached to the heart was used to prepare cryo-sections. Cryo-sections (8 μm) were cut, collected, and stained with oil red O according to [26] with minor modification. The quantification of stained lipids was determined by calculating the positive area in comparison to the total cross-sectional vessel wall area by using Image-Pro Plus software 4.5 (Media Cybernetics). The percentage was calculated from five sections for each animal.

### 2.12. Lipid Peroxidation (Thiobarbituric Acid-Reactive Substances Assay)

Thiobarbituric acid-reactive substances (TBARS) assay was assessed as an index of lipid peroxidation according to [27]. More details in the Supplementary Materials. 

### 2.13. Carbonylated Proteins

Carbonylated proteins were evaluated in plasma and heart tissue homogenate. Plasma or heart tissue protein extracts were quantified by BCA. Twenty micrograms of each sample were denatured by 6% SDS and derivatized by 15-min incubation with 2-4 dinitrophenyl hydrazine (DNPH; Sigma-Aldrich, Italy) at room temperature. More details in the Supplementary Materials.

### 2.14. Western Blot Analysis

Hearts were homogenized in lysis buffer containing 50 mM Tris-HCl pH 8.0, 150 mM NaCl, 1 mM EDTA, 0.5% Triton X-100, and Complete Protease Inhibitor (Roche), and the homogenate was incubated on ice for 30 min. More details in the Supplementary Materials. 

### 2.15. Statistical Analysis

**Figure 1:**
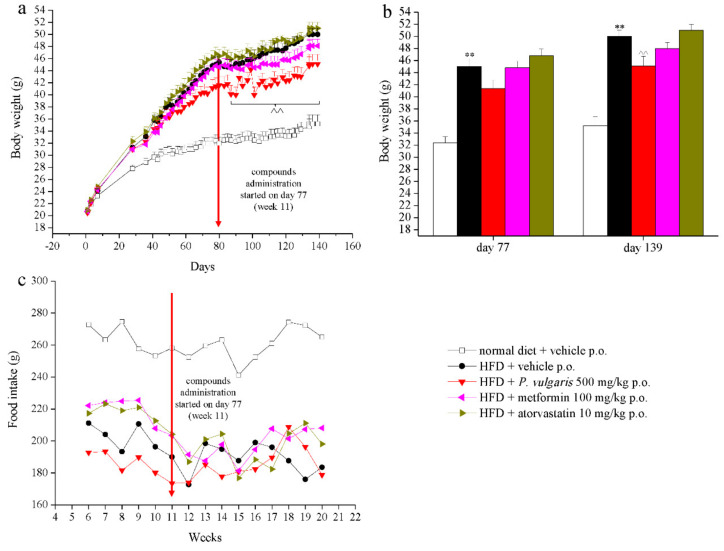
Body weight and food intake. Mice were fed with a normal or a high fat diet (HFD) for 139 days. From day 77 (week 11), the animals were treated daily with vehicle (CMC 1%), *P. vulgaris* extract 500 mg/kg per os (p.o.), metformin 100 mg/kg p.o., and atorvastatin 10 mg/kg p.o. (**a**,**b**) Animals’ body weight evolution, and (**c**) Animals’ food intake evolution. Red arrows point to the beginning of compound administration (day 77, week 11). Each value represents the mean ± S.E.M. of 12 mice per group. ** *p* < 0.01 vs. normal diet + vehicle; ^^ *p* < 0.01 vs. HFD + vehicle.

**Figure 2:**
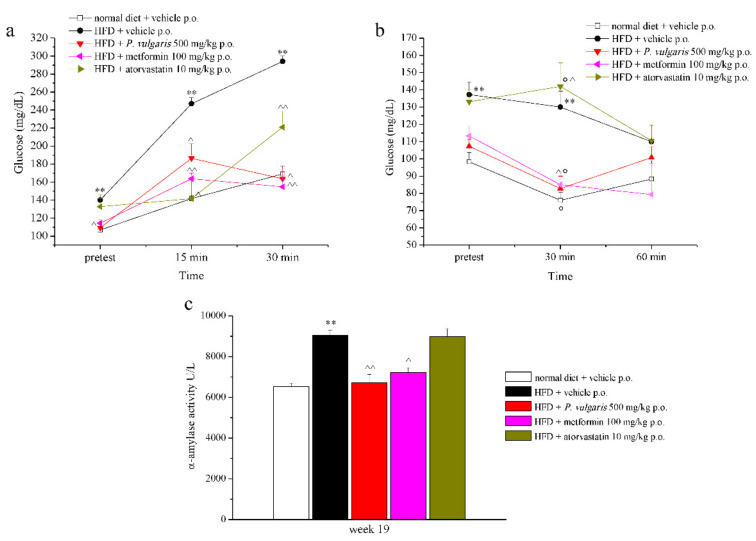
Glucose and insulin tolerance and α-amylase activity. (**a**) Effects of HFD and treatments on glucose tolerance test at week 19; blood glucose was measured before the test (pre-test), and 15 and 30 min after the test. (**b**) Effects of HFD and treatments on insulin tolerance test at week 19. Glucose blood dosages were performed before the test (pre-test), and 30 and 60 min after the test. (**c**) Effects of HFD and treatments on the activity of α-amylase (U/L) in plasmatic sample from animals at week 19. Each value represents the mean ± S.E.M. of 12 mice per group. ** *p* < 0.01 vs. group normal diet + vehicle; ^ *p* < 0.05 and ^^ *p* < 0.01 vs. HFD + vehicle; ° *p* < 0.05 vs. pre-test of the same group.

Measurements were performed on a total of 12 animals for each group, analyzed in 2 different experimental sets (each experimental set was conducted on 6 animals) by researchers blinded to the treatment procedure. Results were expressed as means ± S.E.M. (standard error of mean) of the two experiments and the analysis of variance was performed by one-way or two-way ANOVA tests (Figure 1, Figure 2 and Figure 4). A Bonferroni’s significant difference procedure was used as a post hoc comparison, and *p*-values less than 0.05 were considered significant. Data were analyzed using the “Origin 8.1” software (Northampton, MA, USA).

## 3. Results

### 3.1. Treatments, Body Weight, and Food Intake

C57BL/6 mice were fed with either a normal diet or a high fat diet (HFD) for 19 weeks. Throughout all this time, many parameters were periodically assessed: Body weight, food intake, hematic levels of glucose, triglycerides, and total cholesterol. From the eleventh week on, the mice fed with a high fat diet were divided into three groups and treated with *P. vulgaris* extract 500 mg/kg, metformin 100 mg/kg, and atorvastatin 10 mg/kg. Control groups of both types of feeding were challenged with the vehicle alone (CMC 1%). The drugs, the food extract, and the vehicle were administered daily *per os* (p.o.), 30 min before the dark phase of the circadian light/dark cycle of mice in the animal facility.

Figure 1a,b shows the effects of diet and treatments on body weight of each experimental group from the beginning of the feeding, with a normal or a high fat diet, until the end of the experiment. On day 0, the body weight mean was homogeneous among all groups, but from day 28 a significant rise was evident in all groups fed with a HFD diet, with respect to the group fed with standard diet (31.3 ± 0.7 g vs. 27.8 ± 0.6 g, respectively). The body weight increase of HFD animals remained significant compared to control animals, from the first compounds challenge until the last experimental week. However, *P. vulgaris* extract administration induced a statistically significant weight reduction in HFD mice between day 85 (week 12) and day 139 (week 19) (Figure 1b), corresponding to an average 5 g loss in comparison with non-treated animals (47.3 ± 0.36 g vs. 42.5 ± 0.31 g, respectively). Metformin treatment pointed out a reducing trend in body weight, but eventually it did not reach a statistical significance, while animals given atorvastatin did not show a different weight in comparison to the HFD diet control group (Figure 1b).

Food intake was monitored weekly between the sixth and the 20th weeks (Figure 1c). The normal diet fed group consumed more food than the HFD animals, a trend which did not change throughout the whole experiment. Neither an increase nor a decrease in food intake was observed after week 11, the beginning week of treatments.

### 3.2. Hematic Metabolic Parameters

By this time, blood dosages of total cholesterol, triglycerides, and glucose were measured twice a month. Before the administration of the substances, the above-mentioned hematic parameters were significantly augmented in the high fat diet-fed animals, compared to the control animals (Supplementary Table S1).

In week 13, after two weeks of treatment, *P. vulgaris* extract and atorvastatin reduced total cholesterol levels by 24% and 30%, respectively. This result was constant with statistical significance until the nineteenth week (Table 1). During these last seven days, HDL and LDL levels were also measured. The fat-enriched feeding did not alter HDL values in comparison to control animals, while it increased LDL levels, which were lowered by each treatment. The highest efficacy was reached by *P. vulgaris* extract treatments, which almost halved them.

Triglycerides were lowered from the first challenge only by atorvastatin (by 22%), while *P. vulgaris* and metformin administrations began to be effective from week 15, until week 19 and week 17, respectively, inducing results similar to those obtained by atorvastatin injections (Table 1). As to hematic glucose levels, they were reduced by the administration of *P. vulgaris* extract and metformin, by about a fifth and a sixth, respectively. Atorvastatin appeared to be non-effective (Table 1).

### 3.3. Glucose and Insulin Tolerance Test

The results of the glucose tolerance test (GTT) performed at week 19 are displayed in Figure 2a. Glucose tolerance is shown by the HFD control animals, whose maximum glycaemic peak, reached 30 min after sugar administration (1 g/kg p.o.), was 294.0 ± 5.9 mg/dL. A reduction of hematic glycaemia was observed in the treated groups, at all times of the experiment. Indeed, 30 min after the beginning of the test, glucose levels of *P. vulgaris* extract, atorvastatin, and metformin-treated mice were similar to those of normal diet control mice, while this value almost doubled in non-treated HFD group. Next to the glucose tolerance test, the insulin tolerance test (ITT) was also performed at week 19 (Figure 2b). High fat diet-fed animals were resistant to insulin injection (1U/kg i.p.), as demonstrated by stable glycaemic values 30 and 60 min following the administration, in comparison with the pre-test. A slight, non-significant delayed response to insulin of HFD animals can be highlighted. The groups treated with *P. vulgaris* extract and metformin were sensitive to the hormonal hypoglycaemic activity, as explained by the reduced glycaemic values with respect to both the pre-test and the 30 min dosages of the HFD controls. In the latter comparison, a one third reduction was observed, while in the first comparison, there was a 22% and a 25% decrease due to *P. vulgaris* extract and metformin, respectively. Atorvastatin treatment did not lower the tolerance to the insulin induced by high fat diet. The evaluations of GTT and ITT were performed on fed animals according to the literature which shows that the measure of glucose concentrations in fed state mice is informative as well as the measure performed in fasted animal [28]. The values of the area under the curve (AUC) for GTT and ITT were reported in Supplementary Table S2. The HOMA-IR values were also calculated and are reported in Supplementary Table S3.

### 3.4. Plasma Enzymatic and Oxidative Parameters

During the last experimental week, insulin and ghrelin plasmic levels were measured. Insulin was higher in HFD non-treated with respect to control animals. *P. vulgaris* extract and metformin lowered that value in a significant way by about 80% (Table 2). Ghrelin plasmic values also appeared to be altered in animals on high fat diet with respect to the control animals (10.5 ± 3.4 ng/mL vs. 38.6 ± 5.8 ng/mL, respectively), and under treatment there was a trend to increase, but with no significance (Table 2). Then, plasmatic alpha-amylase enzymatic activity was measured (Figure 2c). The fat-enriched diet positively modulated enzyme activity with respect to the control levels, as it was almost unvaried in *P. vulgaris* extract and metformin-treated groups. Atorvastatin administration was not able to lower the high values induced by the high fat diet. 

**Figure 3:**
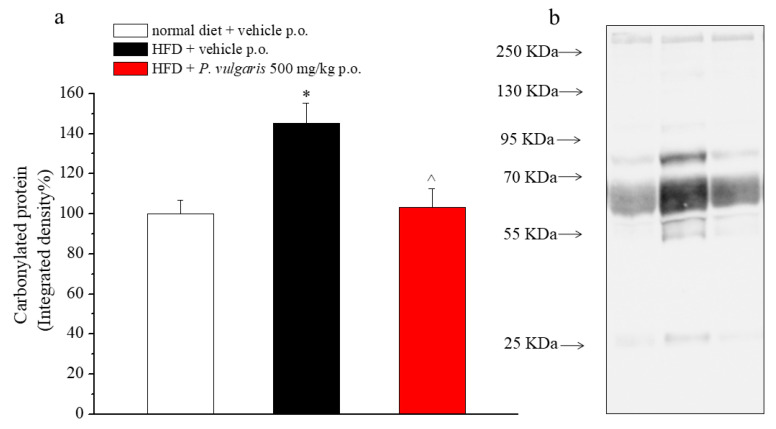
Plasmatic oxidation. Effects of HFD and treatments on the levels of carbonylated proteins in plasma (week 19). Protein oxidative damage was quantified by immunoblot. (**a**) Densitometric analysis and (**b**) representative Western blot are shown. Ponceau-stained membranes were used as loading control. Results are expressed as % of control group (normal diet + vehicle; 100%). Each value represents the mean ± S.E.M. of 12 mice per group. * *p* < 0.05 vs. normal diet + vehicle; ^ *p* < 0.05 vs. HFD + vehicle.

Another parameter measured from plasma was protein carbonylation (Figure 3), which was evaluated as integrated density (Figure 3a) from Western blot membranes (Figure 3b). Plasma from HFD mice had a 45% increase of carbonylated proteins than plasma from control animals. That value was reduced nearly to the control levels by chronic treatment with *P. vulgaris* extract.

### 3.5. Behavioral Measurements

On week 19, behavioral measurements were also made, in order to highlight motorial and neurological alterations due to the fat-enriched diet. 

**Figure 4:**
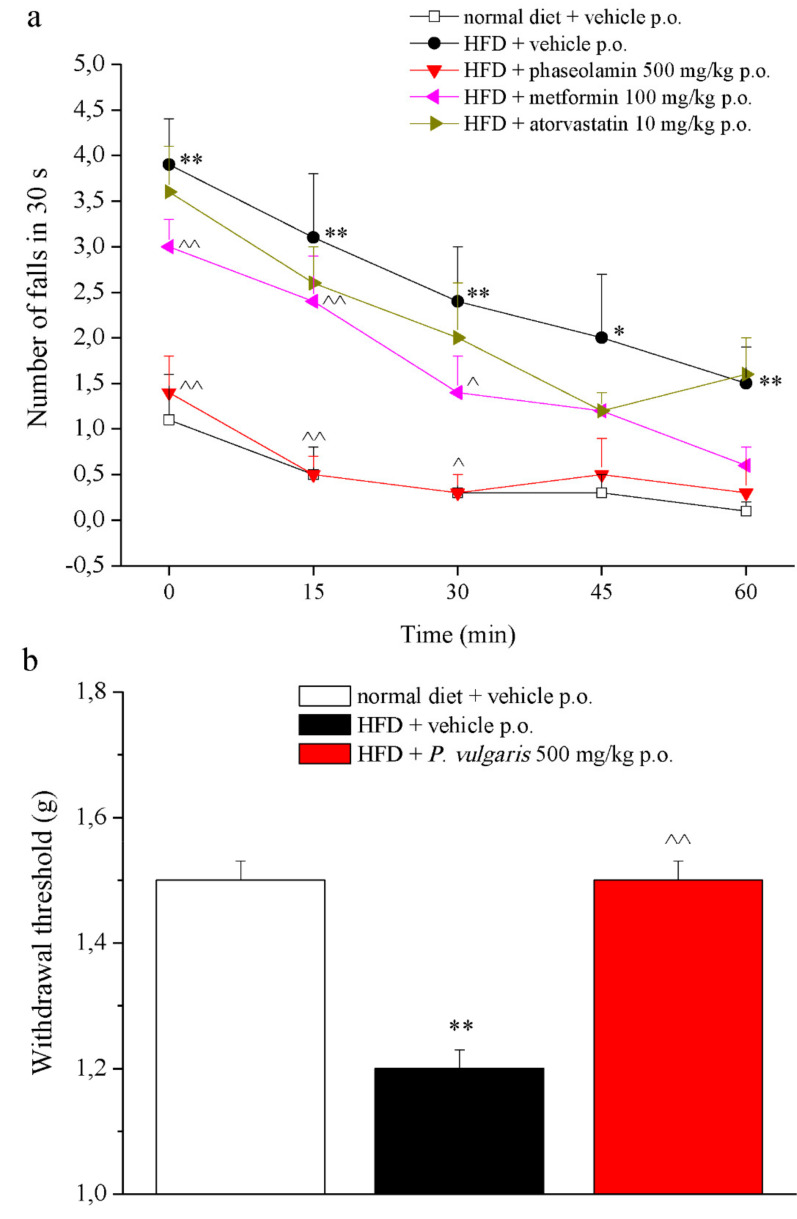
Behavior. (**a**) The integrity of the animals’ motor coordination (week 19) was assessed using a rota-rod apparatus. Rats were placed on a rotating rod (10 rpm) for 30 s every 15 min for 1 h. The number of falls was counted. (**b**) The pain threshold was measured by the Von Frey test to assess the response evoked by a mechanical non-noxious stimulus. Each value represents the mean ± S.E.M. of 12 mice per group. * *p* <0.05 and ** *p* < 0.01 vs. normal diet + vehicle; ^ *p* < 0.05 and ^^ *p* < 0.01 vs. HFD + vehicle.

The Rota Rod test (Figure 4a) was used to evaluate motor coordination. The HFD animals fell from two to three times more than the control animals throughout the one-hour exercise. Among all the treatments, only *P. vulgaris* extract had restoring effects, making mice fall the same number of times as the control animals. The Von Frey test evaluated mechanical allodynia evoked by a non-noxious stimulus (Figure 4b). As shown by the graph, HFD mice demonstrated a significantly lowered nociceptive threshold with respect to the control animals (1.2 ± 0.03 g vs. 1.5 ± 0.03 g, respectively). Again, *P. vulgaris* extract treatment reverted this pain condition to the control threshold. Other two behavioral tests, the plantar test (Supplementary Table S4) and the elevated plus maze test (Supplementary Table S5), did not show significant alterations between the two control groups.

### 3.6. Ex Vivo Analysis

At the end of the nineteenth week, the animals were sacrificed and some of their organs were explanted for subsequent ex vivo analysis. 

**Figure 5:**
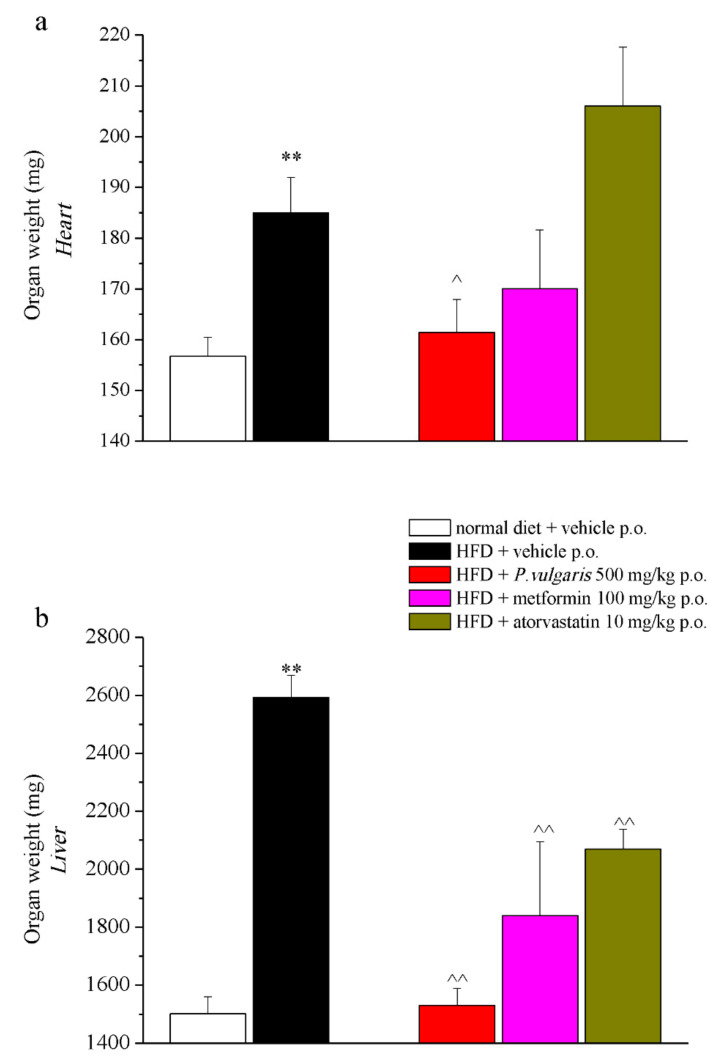
Organ weight. An week 19, after in vivo examinations, organs were collected and weighed. (**a**) Heart; (**b**) liver. Each value represents the mean ± S.E.M. of 12 mice per group. ** *p* < 0.01 vs. normal diet + vehicle; ^ *p* < 0.05 and ^^ *p* < 0.01 vs. HFD + vehicle.

The heart, the liver, and the brain were weighed: the heart and the liver of non-treated HFD mice had a higher weight than the organs of mice fed with a normal diet (Figure 5a,b). The administration of *P. vulgaris* extract reduced the weight gain of the heart and liver ensuing the fat-enriched diet, reaching a weight highly close to that of the control mice organs. Tissue weight of the liver, but not the heart, was brought down also by metformin and atorvastatin administrations, but in a less remarkable way than the food extract did: the latter reduced the weight by 40%, the drugs only by 30 and 20 percent, respectively (Figure 5b). As to the brain, no significant alterations were observed when comparing tissue weight of the different experimental groups (Supplementary Figure S1). 

**Figure 6:**
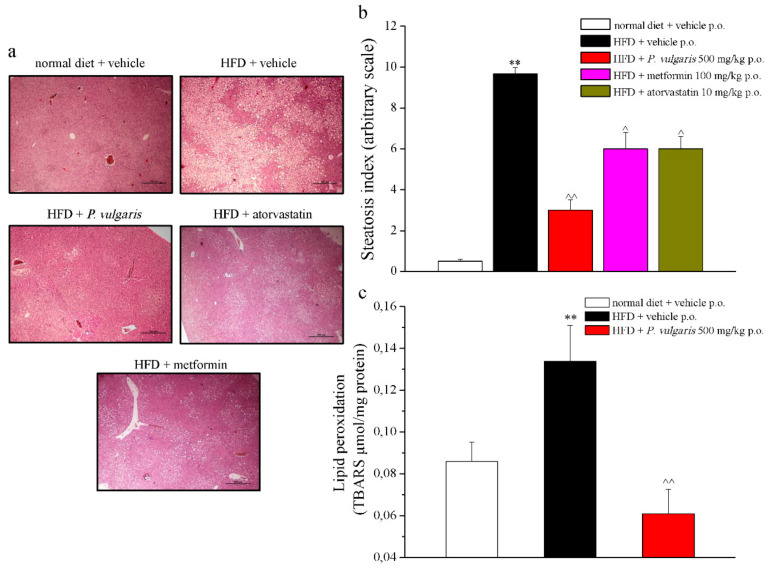
Liver damage parameters. (**a**) Representative images of paraffin-embedded liver sections (5 µm thickness), stained with hematoxyllin-eosin, 4X magnificated. (**b**) Quantitative expression of steatosis index (graded ‘0’ to ‘10’ based on the average percent of fat-accumulated, 0 <5%, 10 >75%). (**c**) Oxidative alteration. Lipid peroxidation was evaluated measuring TBARS (μmol/mg protein). Week 19. Each value represents the mean ± S.E.M. of 12 mice per group. ** *p* < 0.01 vs. normal diet + vehicle; ^ *p* < 0.05 and ^^ *p* < 0.01 vs. HFD + vehicle.

Other histological and quantitative examinations were performed on the explanted tissues. As is shown in Figure 6a, the hypercaloric diet caused a diffuse steatosis in the hepatic parenchyma, not present in the liver of normal diet-fed mice. The pathological state was also expressed in numbers by the steatosis index, as 9.66 ± 0.33 vs. 0.5 ± 0.1, referring to HFD and control mice, respectively (Figure 6b). Another liver damage parameter to be evaluated was lipid peroxidation, expressed as micromoles of thiobarbituric acid reactive substances (TBARS) per milligrams of protein present in the sample. Feeding with fat-rich food caused a 40% more oxidative state of the lipidic component in comparison to the control, while *P. vulgaris* extract treatment avoided the lipid peroxidation increase, whose state was maintained similar to the control levels (Figure 6c). 

**Figure 7:**
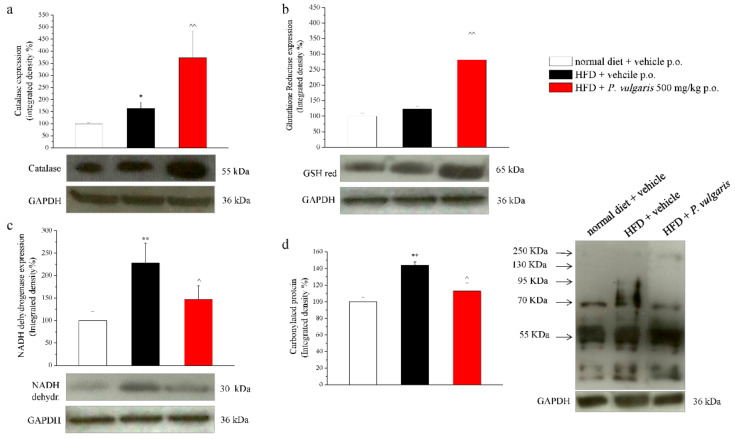
Cardiac damage parameters. Tissue homogenate was processed by Western blot in order to evaluate the protein expression of (**a**) catalase; (**b**) glutathione reductase; (**c**) NADH dehydrogenase; and (**d**) carbonylated proteins. Each parameter is reported as representative blot and densitometric analysis. GAPDH normalization was performed for each sample. Results are expressed as % of control group (normal diet + vehicle; 100%). Week 19. Each value represents the mean ± S.E.M. of 12 mice per group. * *p* < 0.05 and ** *p* < 0.01 vs. normal diet + vehicle; ^ *p* < 0.05 and ^^ *p* < 0.01 vs. HFD + vehicle.

In Figure 7, four cardiac damage parameters are illustrated: the expression of catalase enzyme (7a), glutathione reductase enzyme (7b), NADH dehydrogenase enzyme (7c), and the level of protein carbonylation (7d). Catalase enzyme expression, involved in the detoxification of hydrogen peroxide, appeared to be increased by 62% in the hearts of HFD mice and by 200% in those of HFD mice challenged with *P. vulgaris* extract, in comparison with the control hearts. As to glutathione reductase, which reduces oxidized endogen glutathione, its expression was positively modulated by a high fat diet and by *P. vulgaris* extract administration. In the graph, the integrated density calculation showed a 23% increased expression in the hearts of HFD mice and an 180% increase in those of HFD *P. vulgaris* extract-treated mice. The third analyzed enzyme was NADH dehydrogenase, also named Complex I, which transfers electrons from NADH to an acceptor in the mitochondrial respiratory chain. The results show that the prolonged alimentation with high fat food stimulated a significative increase of the enzyme expression with respect to the control. The integrated density calculated on heart samples of the HFD animals increased by 120%, compared to the control. A decreasing trend, but not statistically significant, was observed when the animals were challenged with *P. vulgaris* extract. Eventually, protein carbonylation dosage gave a measure of the oxidative stress state of the heart. Carbonylated proteins were 45% more in the heart of the HFD animals, compared to the control animals, a condition which was not reduced in a significant way by *P. vulgaris* extract administration.

**Figure 8:**
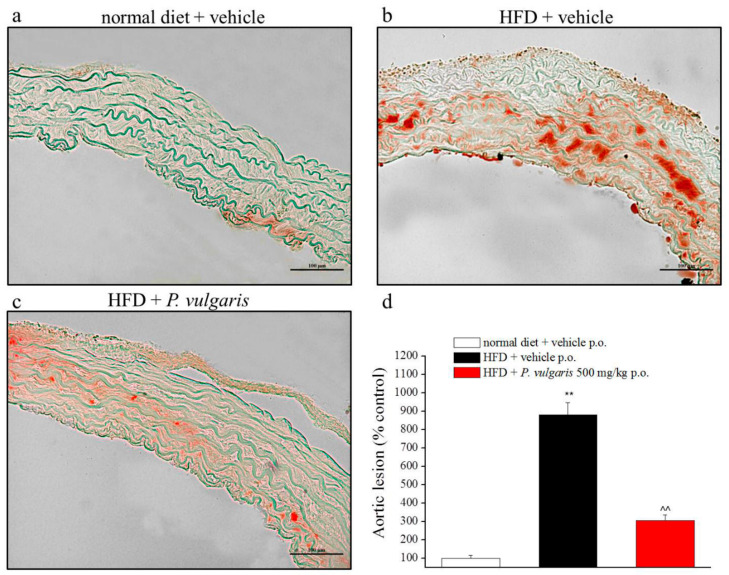
Vascular damage. (**a**–**d**) Evaluation of fatty streak infiltrations in aortic tissue of animals an week 19. (**a**,**b**,**d**) Representative images of aortic arch sections stained with Oil-Red O, 20X magnificated. (**d**) Quantitative determination of lesion extension, calculated as the positive area in comparison to the total cross-sectional vessel wall area. Aortic damage is expressed as percentage in comparison to control samples (set as 100%). ** *p* < 0.01 vs. normal diet + vehicle; ^^ *p* < 0.01 vs. HFD + vehicle.

Next to the cardiac parameters, vascular system condition was also controlled. Oil Red O staining made it possible to evaluate lipidic infiltrations in the aortic muscular layers (Figure 8a–d). HFD feeding extensively augmented lipid infiltration in the aortic tissue, rather than normal alimentation did (879 ± 66% vs 100 ± 15%, respectively). Sustained *P. vulgaris* challenges more than halved the lipid deposit built up by high calories feeding. 

**Figure 9:**
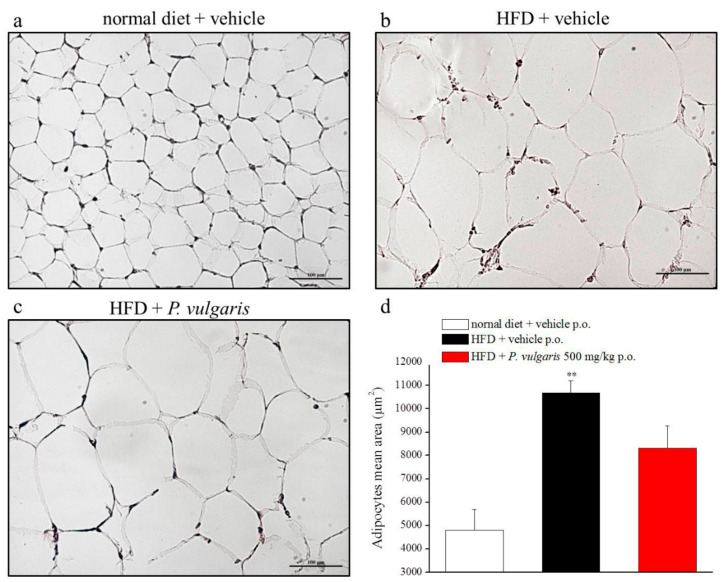
Adipose tissue damage. (**a**–**d**) Effects of diet and *P. vulgaris* treatment on white adipose tissue. (**a**–**c**) Representative images of subcutaneous adipose tissue sections stained with hematoxylin-eosin, 20X magnificated. (**d**) Quantitative determination of adipocytes mean area (µm^2^) following HFD or *P. vulgaris* administration. Each value represents the mean ± S.E.M. of 12 mice per group. **p < 0.01 vs. normal diet + vehicle.

Histological analysis was also made on epidydimal white adipose tissue. The photomicrographs represent the effect of fat-enriched alimentation on the area of the adipocytes: The HFD adipocyte mean area doubled the control mean area and *P. vulgaris* extract treatment did not lower significantly the adipocyte enlargement (Figure 9a–d). 

Morphologic analysis of renal tissue did not highlight any pathologic alteration between the two differently fed group of animals: Both glomerular and mesangial regions showed unvaried area measures with respect to the control (Supplementary Figure S2). 

## 4. Discussion

The relevance of metabolic syndrome is due to the severity of the disorders that it involves: Hyperglycaemia, insulin resistance, dyslipidaemia, obesity, atherogenic events, and hepatic steatosis. All these hallmarks can easily lead to the onset of type 2 diabetes and cardiovascular problems. Therefore, there is a real need for drugs able to target one or more of these disturbs, with few minor side effects.

In the present study, we successfully produced a murine model of metabolic syndrome, in which we investigated the protective effects of a standardized extract from *P. vulgaris* seeds. Its activity was compared with two reference drugs, metformin and atorvastatin, that are used in clinical for the management of hyperglycaemia and hypercholesterolemia, respectively. We obtained highly encouraging results, as we demonstrated that the natural substance equalled and even sometimes outdid the reference drug beneficial effects. The originality of our work consists in the evaluation of the effect of an eight week daily treatment with a *P. vulgaris* extract on all hallmarks of metabolic syndrome, ranging from the effect on blood glucose, triglycerides and cholesterol levels to the effect on pain and motor alteration to the ex vivo analysis. We investigated the protective effect of *P. vulgaris* extract repeated treatment on liver steatosis, on vascular damage, and on oxidative stress, highlighting that the mechanism of action of *P. vulgaris* is due to its constituent phaseolamin, an alpha-amylase inhibitor. The onset of the syndrome was induced by high fat diet (HFD) feeding, containing 60% fats out of the total calories, composed by animal fat and sucrose. The first induces metabolic disorders more effectively than vegetable fat does, while the second exacerbates the correlated negative outcomes, above all dyslipidaemia [29].

We firstly showed that *P. vulgaris* extract treatment significantly prevented the weight gain in HFD mice, even more than treatment with the reference drugs. Several works on *P. vulgaris* extract reported this anorexigenic effect, on diabetic and healthy rats, following acute or chronic administration in ranging doses of 50–500 mg/kg. In such cases, it was supported by a concomitant reduction in food intake [30,31], a condition which we did not observe. A cholecystokinin-mediated mechanism was suggested [30] to explain the food intake decrease, nevertheless Reverri et al. [32] showed that the increased plasmatic concentration of cholecystokinin in human subjects treated with a meal enriched with black bean did not produce the expected heightened satiety.

Weight loss can be explained as independent of food intake: Fat reserves of HFD mice could have been mobilized due to reduced total energy in form of glucose, whose plasmatic level was lowered under treatment [17,33,34]. Some decades after its discovery, the alpha amylase inhibitor-1 phaseolamin was tested for anti-obesity starch-blocker effects on humans: Even if a significant weight loss in obese and healthy subjects treated with the commercial αAI1 extract Phase 2^TM^ was reported, in other trials different commercial phaseolamin-based starch-blockers did not influence body weight. Therefore, one could state that the anorexigenic effect is probably related to the maintenance of elevated enzymatic anti-amylase activity and purity level of the compound, which in turn depends on extraction and preparation methods of the starch-blocker commercial product [7].

We demonstrated that chronical administration of a *P. vulgaris* extract, containing alpha-amylase inhibitor and phyto-haemagglutinin, had alleviating effects on three hallmarks of metabolic syndrome: Hypercholesterolemia, hypertriglyceridemia, and hyperglycaemia. *P. vulgaris* extract behaved as a hypocholesterolemic agent on HFD mice soon after two weeks of treatment: It produced results similar to those obtained by the reference drug atorvastatin; moreover, it was the most efficient compound for decreasing LDL values during the last experimental week, suggesting an increasing protective effect. Similar studies on the bioactive natural compound, carried out on diabetic rats, did not highlight any significant change in cholesterol levels [11], but a clinical open-label study on ten overweight and hypercholesterolemic patients underlined a remarkable decrease of total cholesterol following a prolonged treatment with granular food containing the *P. vulgaris* dry extract mixed with other bioactive compounds [35]. A study conducted by Nunez-Aragon and colleagues indicated that total hydrolysates and <1 kDa fractions from *P. vulgaris* showed antihyperglycemic activity, and this activity was due to intestinal glucose absorption and α-glucosidase enzyme inhibition and was safe up to 5000 mg kg^−1^. Bean-derived protein hydrolysates and fractions may therefore have the potential to be used in functional foods, dietary supplements, or pharmaceutical preparations for the prevention and/or treatment of type 2 diabetes [36].

The beneficial effect of *P. vulgaris* extract treatment on blood triglycerides outdid the hypolipidemic activity of metformin and equalled that of atorvastatin, effective a couple of weeks before the extract and metformin. 

The kidney extract was protective against hyperglycaemia, a result consistent with many studies on rats (diabetic or healthy, fed with normal or starch-enriched diet), where different quantities of the α-amylase inhibitor, chronically administrated from 10 to 20 days, induced a significant reduction of glucose blood levels at the end of the prolonged treatment. In addition, when acutely administrated, this lowering effect was observed on the post-prandial glycaemia levels, even if not always statistically significant [17,33,34]. The efficacy on post-prandial hyperglycaemia was confirmed in humans by a study of Spadafranca and her collaborators on healthy men and women, who were given a tablet of *P. vulgaris* extract (containing both α-AI and phytohaemagglutinin; the same used in the present study) to be ingested before a standardized meal [37]. Obesity is not only a matter of weight gain, but behind that there are many other factors which predispose to diabetes and cardiovascular diseases. A low-grade chronic inflammation ensues the pro-inflammatory activity of obesity-induced hypertrophied adipocytes and inflammatory cells, and along with increased free fatty acids they alter insulin receptor pathway, till its inhibition [38]. In our study, the HFD provoked an impaired response to both glucose and insulin, confirming the two typical resistance forms of metabolic syndrome. We showed that *P. vulgaris* extract treatment significantly alleviated both tolerances; metformin showed a similar profile, whereas atorvastatin was effective in preventing the altered response to glucose load only. Insulin hormone is crucial in glucose and lipid metabolism and it is also related to the satiety pathway of central and peripheral nervous system: A hampered response of the nervous insulin receptors was reported to be related to appetite control, leading to hyperphagia and obesity. In addition, chronic hyperinsulinemia generally occurring in obese patients appears to function as a compensatory strategy of our organism, in order to restore energy reserves and to limit weight gain [39]. In our experiments, HFD mice at the 19th week of the enriched diet had high insulin plasmatic levels remarkably lowered by *P. vulgaris* extract and metformin treatment, in an almost identical way. 

Ghrelin is another hormone thought to have a prominent role in obesity and metabolic syndrome. Ghrelin is a stomach-derived peptide hormone, produced by the endocrine cells of the oxyntic mucosa, which regulates appetite. Its levels increase before a meal and decrease soon after ending a meal; this post-prandial suppression communicates satiety and food reward to the central nervous system (CNS). Although fasting ghrelin levels of obese individuals are lower than those of lean people, the absence of a physiological decrease after a meal explains their non-stop eating behavior [37,39]. In her paper, Spadafranca and her collaborators sustained the hypothesis of a link between ghrelin suppression and *P. vulgaris* extract administration [37]. In our study we cannot confirm this: Non-treated HFD mice had low ghrelin levels, but hormonal secretion appeared not to be influenced by the prolonged *P. vulgaris* extract treatment; ghrelin levels increased without reaching a statistical significance. 

The results of the present study stressed another mechanism, the ability of *P. vulgaris* extract to negatively modulate enzymatic activity of pancreatic alpha-amylase, which in extract-treated HFD mice almost reached the levels of controls. Phaseolamin is, indeed, commonly recognized as a starch blocker, a compound which slows down the digestion of complex carbohydrates, and thus gastric emptying as well, due to the inhibition of α-amylase enzymatic activity [7,37,40].

A growing body of literature links prediabetes, obesity, and metabolic syndrome to the risk of diabetic peripheral neuropathy as well as cryptogenic sensory peripheral neuropathy [41]. On this basis, we focused on neurological impairments; in the HFD group we observed a decreased pain threshold, highlighting a condition that resembles mechanical allodynia, as well as altered motor coordination. Interestingly, these alterations were reverted only in the *P. vulgaris* extract treated-group. This point could be explained since phytotherapy is based on the combined action of a mixture of constituents able to offer a multiple approach to the multi-factorial nature of the metabolic syndrome. The bioactivity of crude drugs or vegetal extracts is a summation of antagonistic and/or synergistic effects on bioavailability, cellular transport processes, compound metabolism, and pharmacodynamic mechanisms, which could explain the positive effect of *P. vulgaris* extract also on pain threshold and motor alterations induced by HFD. On the contrary, metformin and atorvastatin, two drugs with a specific function and mechanism of action, are ineffective. No effects on thermal hyperalgesia or anxiety were recognized in HFD-fed animals. A similar annotation of metabolic syndrome fallouts on neurological behavior was published in a work by Takase and his colleagues, who observed that HFD evoked motorial disfunctions in mice, with no influences on anxiety [42].

A fat-enriched diet considerably affects the liver, the organ dedicated to lipid and lipoprotein metabolism, in addition to—and because of—the onset of glucose and insulin resistance. The increased circulating free fatty acids overload the liver with fat and cholesterol, causing hepatotoxicity, a state of dyslipidemia, excess fat deposing and cardiovascular diseases. In particular, the fatty diet and insulin resistance cause a switch from the physiological mitochondrial lipid oxidation to peroxidation of long chain fatty acids, which promotes lipotoxicity and extraordinary release of reactive oxygen species (ROS) [43].

The high fat diet caused a gain weight of liver and heart in the HFD mice, but it was significantly lowered by treatment with *P. vulgaris* extract. Also, the other steps of on-going metabolic syndrome were confirmed. Histological samples from the HFD group clearly showed hepatosteatosis, hepatic lipid peroxidation, and lipid accumulation in the cardiovascular system, such as the fatty streaks in the aorta. These disease-related negative consequences were reverted by *P. vulgaris* extract challenge. 

As mentioned above, the excessive generation of ROS, when not counterbalanced by the antioxidant mechanisms, is a pathological condition which is dramatically augmented in metabolic syndrome, thus inducing the antioxidant defense system to change in relation to the new state. Protein carbonylation in the plasma, normally increased in a fat diet, was significantly reduced in *P. vulgaris* extract -treated mice, showing a protective role of the compound against this oxidative stress consequence. Conversely, it was not sufficient to protect the HFD heart, whose carbonylated proteins were not significantly diminished by the same treatment. 

Aiming to study possible redox detoxifying mechanisms, we evaluated the expression levels of cardiac antioxidant enzymes. Catalase and GSH reductase were considerably more expressed in HFD hearts, probably obeying to a pathophysiological compensatory mechanism, to face the elevated cardiac ROS levels. *P. vulgaris* extract treatment raised the levels of all the antioxidant enzymes, except for NADH dehydrogenase which was slightly reduced. 

## 5. Conclusions

The present standardized *P. vulgaris* extract counteracts molecular, biochemical, and behavioral alterations induced by HFD in a clinically-relevant model of metabolic syndrome. The wide range of activity, as well as the well assessed safety profile [40], makes *P. vulgaris* extract an intriguing candidate for a clinical use.

## Figures and Tables

**Table 1:** Hematic parameters.

		Group				
	Week	Normal Diet + Vehicle	HFD + Vehicle	HFD + *P. vulgaris*	HFD + Metformin	HFD + Atorvastatin
**Glucose (mg/dL)**	*13*	101.0 ± 3.4	139.3 ± 6.9 *	103.0 ± 5.1^	132.0 ± 9.9	133.0 ± 6.2
	*15*	99.0 ± 10.1	149.5 ± 3.9 **	103.0 ± 8.0^^	113.3 ± 7.4 ^	112.8 ± 7.1 ^
	*17*	103.3 ± 6.4	138.5 ± 9.1 *	127.8 ± 2.0 ^^	121.0 ± 5.9 ^	146.3 ± 5.5
	*19*	99.3 ± 4.6	137.3 ± 9.4 **	112.0 ± 4.4 ^	116.3 ± 5.7 ^	137.3 ± 14.0
**Triglycerides** **(mg/dL)**	*13*	62.0 ± 6.9	132.3 ± 11.3 *	123.0 ± 18.0	105.0 ± 14.6	102.0 ± 9.0 ^
	*15*	72.3 ± 13.6	135.3 ± 6.0 **	86.5 ± 10.7 ^	93.3 ± 10.4 ^	103.3 ± 9.4 ^
	*17*	78.4 ± 2.7	136.8 ± 2.3 **	105.3 ± 7.8 ^^	113.8 ± 5.8 ^	108.8 ± 7.2 ^^
	*19*	75.8 ± 11.0	133.5 ± 7.7 **	107.5 ± 9.3 ^^	118.3 ± 3.7	104.0 ± 3.0 ^^
**Total cholesterol** **(mg/dL)**	*13*	<100	133.7 ± 4.4 **	104.3 ± 3.5 ^^	128.6 ± 8.2	105.0 ± 6.0 ^^
	*15*	105.0 ± 5.0	135.8 ± 1.3 **	103.0 ± 2.3 ^^	118.3 ± 7.2	105.0 ± 4.3 ^^
	*17*	107.0 ± 1.2	147.0 ± 2.8 **	123.0 ± 6.4 ^^	139.3 ± 12.5	109.5 ± 5.0 ^^
	*19*	103.8 ± 4.1	147.3 ± 4.1 **	100.5 ± 7.6 ^^	103.9 ± 9.2 ^^	76.4 ± 1.4 ^^
**HDL (mg/dL)**	*19*	51.8 ± 8.5	62.3 ± 7.0	41.7 ± 10.2	59.1 ± 10.5	46.8 ± 9.5
**LDL** **(mg/dL)**	*19*	43.8 ± 6.7	62.3 ± 7.0	38.4 ± 6.3 ^^	46.0 ± 9.4 ^	50.2 ± 3.0 ^^

Effects of HFD and treatments on glucose, triglycerides, and total cholesterol blood concentration at weeks 13, 15, 17, and 19. At week 19, HDL and LDL values were also assessed. Animals were fed with normal or HFD diets from week 1. *P. vulgaris* extract (500 mg/kg), metformin (100 mg/kg), and atorvastatin (10 mg/kg) were administered daily per os, starting from week 11. Each value represents the mean ± S.E.M. of at least 12 mice per group. * *p* < 0.05 and ** *p* < 0.01 vs. normal diet + vehicle; ^ *p* < 0.05 and ^^ *p* < 0.01 vs. HFD + vehicle.

**Table 2:** Insulin and ghrelin levels.

	Group				
	Normal Diet + Vehicle	HFD + Vehicle	HFD + *P. vulgaris*	HFD + Metformin	HFD + Atorvastatin
**Insulin (ng/mL)**	4.2 ± 0.5	46.1 ± 7.3 **	8.9 ± 8.2 ^^	10.2 ± 7.6 ^^	34.9 ± 10.5
**Ghrelin (ng/mL)**	38.6 ± 5.8	10.5 ± 3.4 *	17.0 ± 2.8	13.8 ± 4.3	15.8 ± 2.4

Effects of HFD and treatments on insulin and ghrelin plasma concentration at week 19. At week 19, HDL and LDL values were also assessed. Animals were fed with normal or HFD diets from week 1. *P. vulgaris* extract (500 mg/kg), metformin (100 mg/kg), and atorvastatin (10 mg/kg) were administered daily per os starting from week 11. Each value represents the mean ± S.E.M. of at least 12 mice per group. * *p* < 0.05 and ** *p* < 0.01 vs. normal diet + vehicle; ^^ *p* < 0.01 vs. HFD + vehicle.

## Supplementary Materials

The following are available online at https://www.mdpi.com/2072-6643/11/8/1778/s1, Materials and Methods, Table S1: Hematic metabolic parameters; Table S2: GTT and ITT AUC; Table S3: HOMA-IR; Table S4: Plantar test; Table S5: Elevated plus maze; Figure S1: Brain weight; and Figure S2: Kidney histological evaluation.
