# Correction: Micheli et al. *Phaseolus vulgaris* L. Extract: Alpha-Amylase Inhibition Against Metabolic Syndrome in Mice. *Nutrients* 2019, *11*, 1778

**DOI:** 10.3390/nu18132116

**Published:** 2026-06-29

**Authors:** Laura Micheli, Elena Lucarini, Elena Trallori, Carmen Avagliano, Carmen De Caro, Roberto Russo, Antonio Calignano, Carla Ghelardini, Alessandra Pacini, Lorenzo Di Cesare Mannelli

**Affiliations:** 1 Department of Neuroscience, Psychology, Drug Research and Child Health-Neurofarba-Pharmacology and Toxicology Section, University of Florence, 50139 Florence, Italy; laura.micheli@unifi.it (L.M.); elena.lucarini@unifi.it (E.L.); elena.trallori@unifi.it (E.T.); carla.ghelardini@unifi.it (C.G.); 2 Department of Pharmacy, University of Naples “Federico II” Naples, 80131 Naples, Italy; carme.avagliano@unina.it (C.A.); roberto.russo@unina.it (R.R.); antonio.calignano@unina.it (A.C.); 3 Department of Science of Health, School of Medicine and Surgery, University of Catanzaro, 88100 Catanzaro, Italy; carmen.decaro@unicz.it; 4 Department of Experimental and Clinical Medicine, Anatomy and Histology Section, University of Florence, 50134 Florence, Italy; alessandra.pacini@unifi.it

In the original publication [1], there was a mistake in Figure 3. Inadvertently, during figure preparation, an incorrect image was used as the representative Western blot. The corrected Figure 3 appears below. The authors state that the scientific conclusions are unaffected. This correction was approved by the Academic Editor. The original publication has also been updated.

## Figures and Tables

**Figure 3:**
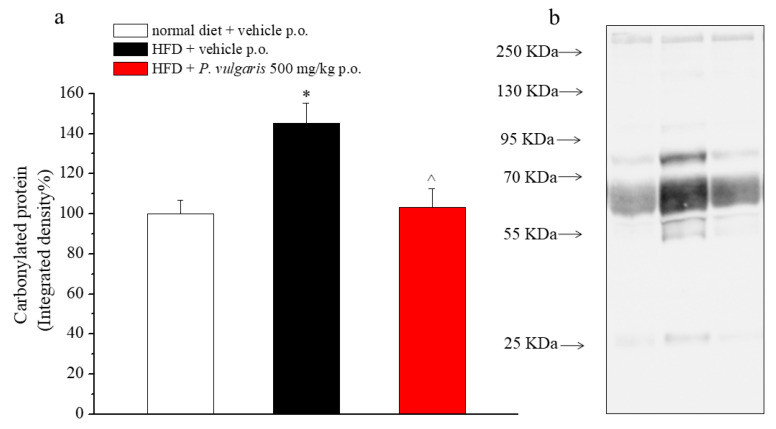
Plasmatic oxidation. Effects of HFD and treatments on the levels of carbonylated proteins in plasma (week 19). Protein oxidative damage was quantified by immunoblot. (**a**) Densitometric analysis and (**b**) representative Western blot are shown. Ponceau-stained membranes were used as loading control. Results are expressed as % of control group (normal diet + vehicle; 100%). Each value represents the mean ± S.E.M. of 12 mice per group. * *p* < 0.05 vs. normal diet + vehicle; ^ *p* < 0.05 vs. HFD + vehicle.
